# Risks and Benefits of Magnesium Sulfate Tocolysis in Preterm Labor (PTL)

**DOI:** 10.3934/publichealth.2016.2.348

**Published:** 2016-05-30

**Authors:** John P. Elliott, John C. Morrison, James A. Bofill

**Affiliations:** 1 Banner University Medical Center, Phoenix, 1111 East McDowell Road, Phoenix, AZ 85005; 2 Department of Obstetrics and Gynecology, University of Mississippi Medical Center, 2500 North State Street, Jackson, Mississippi, 39216

**Keywords:** magnesium sulfate, tocolysis, preterm labor, FDA

## Abstract

The U.S. Food and Drug Administration issued a drug safety communication on 05/30/2013 recommending “against prolonged use of magnesium sulfate to stop preterm labor (PTL) due to bone changes in exposed babies.” In September of 2013, The American Congress of Obstetrics and Gynecologists issued Committee Opinion No. 573 “ Magnesium Sulfate Use in Obstetrics” , which supports the short term use of MgSO_4_ to prolong pregnancy (up to 48 hrs.) to allow for the administration of antenatal corticosteroids.” Are these pronouncements by respected organizations short sighted and will potentially result in more harm than good? The FDA safety communication focuses on bone demineralization (a few cases with fractures) with prolonged administration of MgSO_4_ (beyond 5–7 days). It cites 18 case reports in the Adverse Event Reporting System with an average duration of magnesium exposure of 9.6 weeks (range 8–12 wks). Other epidemiologic studies showed transient changes in bone density which resolved in the short duration of follow up. Interestingly, the report fails to acknowledge the fact that these 18 fetuses were in danger of PTD and the pregnancy was prolonged by 9.6 weeks (e.g. extending 25 weeks to 34.6 wks), thus significantly reducing mortality and morbidity. Evidence does support the efficacy of MgSO_4_ as a tocolytic medication. The decision to use magnesium, the dosage to administer, the duration of use, and alternative therapies are physician judgments. These decisions should be made based on a reasonable assessment of the risks of the clinical situation (PTL) and the treatments available versus the benefits of significantly prolonging pregnancy.

## 1. Introduction

Virtually every publication about preterm birth (PTB) cites the profound impact PTB has on the babies, families and the healthcare system. In Practice Bulletin 127 (June 2012) [1], The American College of Obstetrician and Gynecologists (ACOG) states: “Preterm birth is the leading cause of neonatal mortality and the most common reason for antenatal hospitalization. In the United States approximately 12% of all live births occur before term, and preterm labor (PTL) preceded approximately 50% of these preterm births…preterm births account for approximately 70% of neonatal deaths and 36% of infant deaths as well as 25–50% of cases of long-term neurologic impairment in children”. These statistics magnify the importance of PTL contributing to 50% of PTB's.

When a patient presents to the hospital with regular uterine contractions and cervical change between 20 0/7 weeks and 36 6/7 weeks gestation with intact membranes, a diagnosis of PTL is made. Some factor or factors have caused a response of the myometrium (contractions) creating the risk of PTB. The etiologic causes of PTL include: placental abruption, infection or inflammation, excessive stretch (multiple gestation, etc.), drugs, immunologic reactions, and idiopathic. Tocolytic medications are a therapy of failure. They represent a last attempt to delay or prevent a PTB. In the last five years, The U.S. Food and Drug Administration has issued a “black box” warning pertaining to terbutaline and more recently issued a drug safety communication [2] on 05/30/2013 recommending “against prolonged use of magnesium sulfate to stop preterm labor due to bone change in babies.” This prompted ACOG to issue Committee Opinion No. 573 — “On Magnesium Sulfate use in Obstetrics” (Sept. 2013) [3]. Thus, two drugs (terbutaline and magnesium sulfate) have been removed or restricted by organized medicine and the U.S. government from the clinician's therapeutic choices. In this article, we will examine the evidence about magnesium sulfate and make recommendations about appropriate usage of the drug for tocolysis.

Every decision in medical practice involves a clinician assessing the benefits as well as risks of any intervention. In obstetrics that benefit vs. risk involves both the mother and fetus (es), complicating the decision process. In an insightful article in 2009 [4], Dr. Vintzeleos compared evidence based medicine with reality — based medicine. For several decades, medicine has been moving toward “evidence based medicine” [5]. Sackett [6] defined evidence based medicine as “The use of clinical expertise combined with current evidence to make decisions about the care of individual patients.” This definition implies a combination of published data placed into context by the physician based on his/her experience and applied to the individual patient. Prospective randomized blinded placebo controlled trials are considered the “Gold- Standard” and can be important in assessing discrete interventions or therapies. They can be misleading or biased and somehow the clinical expertise of physician has become diminished. Often the best clinical evidence is open to individual interpretation.

## 2. Efficacy of Magnesium Sulfate as a Tocolytic

The first report about MgSO_4_ as a tocolytic was published by Steer and Petrie in 1977 [7] and was followed by the largest case series in the literature by Elliott in 1983 [8], which provided reality-based outcomes of 355 patients who were treated with MgSO_4_ after transport from another hospital with a diagnosis of PTL. Patients had multiple other morbidities such as amnionitis, placental abruption, premature rupture of membranes, or advanced cervical dilation. In singleton gestations with intact membranes, 76% gained 48 hours before delivery, 51% gained 7 days, and 39% achieved a full term delivery. Remember, the MgSO_4_ was mostly an acute medication not chronic and could not reasonably be expected to be efficacious after the drug was discontinued. Elliott, et al [9], demonstrated the peril of an inadequate therapeutic level of magnesium by comparing the incidence of successful tocolysis at 48 hours with low dose Mg (4 gm loading dose / 2 gm per hour), medium dose (4 gm loading dose and greater than 2 gm per hour maintenance) and high dose protocols (6 gm loading dose and greater than 2 gm per hour maintenance). Low dose Mg was successful in 69.2% of treated patients. Medium dose was successful in 79.2% and 88.7% of the high dose patients were successfully tocolysed for 48 hours or more. It is very clear that the serum mg level for successful tocolysis depends on the dosing of the drug. It is common knowledge that many patients treated for PTL are not truly in PTL. Approximately 60% of patients in studies of tocolytics are not in PTL and this will dilute any possible efficacy, while 20% have either intraamniotic infection or occult placental abruption or advanced PTL which will not respond to any tocolytic drug. This leaves approximately 20% of all PTL patients in studies of tocolytic efficacy who can truly be evaluated compared to those treated with placebo or another drug. These factors result in negative outcomes reported in any tocolytic efficacy trial.

In addition to case series and comparison studies with other tocolytic drugs, there are three prospective, randomized trials of MgSO_4_ tocolysis. Two [10],[11] support the efficacy of MgSO_4_ compared to sedation and bed rest [10] and sedation and hydration [11]. Cox, et al [12] found no difference in time gained in utero for magnesium treated patients compared to control. This study deliberately used doses of magnesium that were sub-therapeutic. The introduction states that “concentrations of 8–10 meq/L were observed to reduce the frequency of in vitro muscle contractions “The purpose of the study was to “determine if clinically safe magnesium concentrations (i.e.those less than 8 Meq/L…) were effective in preventing preterm birth when compared with untreated controls.” The mean serum magnesium concentration for the magnesium group was 5.5 +/– 1.4 Meq/L. This is subtheraputic magnesium levels and unlikely to have a therapeutic benefit. They also considered 8 patients as failures if the drug was discontinued for side effects (all were not serious). The methological errors were egregious [13] and yet it was published, which only creates doubt without scientific rigor. Mercer, et al [14] published an extensive review of magnesium sulfate tocolysis. Based on their analysis of 19 selected studies they concluded that magnesium sulfate was equivalent with all other tocolytics and that no tocolytic drug resulted in improved neonatal outcome. They also stated that “it is appropriate to withhold tocolysis with magnesium sulfate or other agents from women presenting in preterm labor as newborn benefit has not been demonstrated with such treatment.” Unfortunately, this is not helpful to an obstetrician faced with evaluating and treating a colleague pregnant with twins at 24 weeks in PTL, three centimeters dilated. I doubt that Dr. Mercer would withhold magnesium or another tocolytic if this were their wife and their babies. Every obstetrician has successfully used magnesium and other tocolytic drugs in these situations. Success does not occur in every patient, but the clinical experience of the physician and the individual patient circumstances should dictate clinical response.

## 3. Are There Harmful Effects on the Mother?

As with any medication, there are side effects with magnesium sulfate. Pulmonary edema occurs in about 1% of patients [8] and is always associated with co morbidities that affect the cardio-vascular system: multiple gestation, anemia, hypertension, infection or fluid overload. Minor side effects occur in 7% of patients [8] including: nausea, muscle weakness, chest heaviness, and “a flushed feeling.” In one study, Magnesium was discontinued in only 2% of patients for side effects [8]. There has been one case report of maternal osteoporosis with prolonged magnesium sulfate tocolysis [15].

## 4. What About Fetal Benefits of Magnesium Sulfate?

One of the celebrated success stories in obstetrics involves the demonstration by metaanalysis that magnesium sulfate given before anticipated preterm birth reduces the risk of cerebral palsy in surviving infants [16]. ACOG [17] does not endorse any particular regimen of magnesium for such neuro-protection but Dr. James Scott in an editorial [18] recommended a loading dose of 4–6 grams of MgSO_4_ and 1–2 grams per hour for 12–24 hours until delivery or discontinue if delivery does not occur, and restart, if necessary. Reeves, et al [19] recommend a 6 gm loading dose and then 2 gm per hour until delivery or 12 hours have elapsed.

## 5. Risks of Magnesium sulfate for the baby (ies)

Concerns about the safety of magnesium sulfate for tocolysis were raised by Mittendorf, et al [20], who published an interim analysis of their MagNET trial which found an excess mortality in the magnesium treated arm of their study. There were 8 deaths of 46 in the magnesium group compared to 0 of 47 in the other tocolytic arm. The deaths appear to be due to chance rather than an effect of the magnesium (SIDS-4, congenital anomalies-1, severe twin-twin-transfusion syndrome-2, 26 week fetus died at delivery-1, death at 260 days from pneumonia in a 25 week delivery). In a significantly larger study involving over 1000 patients, Crowther, et al [21] found a non-significant decrease in total pediatric mortality in magnesium exposed infants. Farkouh, et al [22] published data from 12,876 newborns exposend to magnesium sulfate for tocolysis and concluded that MgSO_4_ was statistically protective against neonatal mortality.

With regard to neonatal morbidity, Mittendorf, et al [23] published an analysis of the MagNet trial data. This trial was testing the hypothesis that magnesium tocolysis would significantly reduce the incidence of intraventricular hemorrhage (IVH) (Gr III and IV). Lacking enough power in their study population, the authors turned to a composite morbidity/mortality endpoint and even inappropriately included IVH Gr I and II which are not clinically significant to babies. If only IVH of Gr. III or IV is considered, there were 2 babies in the magnesium group and 3 in the other tocolysis group, which is not significant. Review of all the available evidence [9] concludes that magnesium sulfate used for tocolysis is not associated with any excess risk of neonatal death or morbidity.

Maintenance tocolysis with magnesium sulfate has not been reported. Maintenance tocolysis would be the use of tocolytic drugs in appropriate candidates beyond 48 hours. Elliott and Morrison [24] published a review article on the evidence regarding maintenance tocolysis, which usually involves oral therapy with magnesium, beta-agonists, or calcium channel blockers after acute treatment for preterm labor in the hospital. Convincing evidence supporting oral maintenance tocolysis is lacking, but supports the use of other maintenance therapy. This article did find convincing evidence supporting “based on all available evidence subcutaneous administration of atosiban or terbutaline by infusion pump appears to be beneficial as maintenance tocolysis.” The action by the FDA in placing terbutaline with a black box warning was unfortunate and unjustified by the evidence. Anecdotally one author (JE) has utilized prolonged MgSO_4_ for maintenance tocolysis in patients with high order multiple gestations (3, 4, 5, 6 fetuses). This has been done in over 200 cases with duration of therapy of from 1–12 weeks. I have considered it essential in prolonging these pregnancies as no other therapy was effective. I have noted rib fractures in 5 or 6 newborns, all of which resolved on follow up. The incidence may be approximately 1%. The trade off is a much greater gestational age at delivery for the babies.

The FDA safety announcement of 05/30/2013 [2] addressed the issue of prolonged use of magnesium sulfate for preterm labor and potential bone changes in the fetus and/or mother. Data from the FDA's adverse event reporting system identified 18 cases previously described in the literature [25]–[28] where the average duration of in utero exposure to magnesium sulfate was 9.6 weeks (range 8–12 weeks). Yokoyama, et al [25] examined 167 neonates retrospectively including 58 whose mothers received intravenous magnesium sulfate administration > 5 days. In their study, the neonatal levels of magnesium and phosphatase were higher than the controls, whereas calcium levels were lower. Likewise alkaline phosphatase levels were also increased and there were two newborns with bone abnormalities. The calcium and phosphate levels returned to normal within two days. By three weeks the serum alkaline phosphate levels were not different between the two groups and there were no bone issues in the offspring. Wedig, et al [26], reported two cases of triplets who were exposed to long term magnesium that revealed bone fractures at birth in two of the six newborns. They noted that serum calcium and phosphate levels were normal by three days of life with no differences in bone mineral content compared to those without exposure to magnesium sulfate. Bone abnormalities noted in the two patients reported by Wedig improved by 19 days of age and no long term effects were noted. Malaeb et al [27] studied four infants including two twin gestations who were exposed to prolonged treatment with magnesium sulfate for preterm labor. Three of the four infants had abnormal mineralization of long bone metaphysis but no fractures were noted. Finally Kaplan et al [28] studied several multifetal gestations whose mothers were treated with long term magnesium sulfate and found osteopenic changes and higher calcium and phosphate metabolism, which reverted to normal after several days with no fractures or other issues noted. The mechanism of these changes is yet unconfirmed, but it is well known that magnesium crosses the placenta and hypermagnesemia is thought to inhibit calcification of the bone directly as it competes with calcium.

Radiographic changes without fractures or electrolyte changes, have been noted and include decrease bone density, widening of the metaphyseal plates but calcification and costochondral junctions appear normal. There were no fractures and these changes were not apparent after one to two weeks. For example, Nasser et al [29] reported on 78 cases where maternal magnesium sulfate treatment was carried out for > 48 hours. These patients were compared to 77 patients who received magnesium sulfate for less than 48 hours. In the study group, there were 19 twins, six triplets and one quadruplet pregnancy. Unfortunately the mean duration of magnesium sulfate treatment was not reported but the range was 2.5–80 days. Among treated patients, there were two mothers who had osteopenia postpartum with normal calcium levels. Among the 78 neonates there were three with abnormal bone mineralization. Both maternal and fetal changes disappeared in a short time after birth. Schanier et al [30] showed similar findings in his study as neonates had abnormal bone mineral content as noted by ultrasound following several weeks of magnesium sulfate therapy for preterm labor. As above, the changes in the newborn disappeared shortly after birth. In spite of these temporary findings in 18 patients (all of which reverted to normal) among those exposed to magnesium for over 48 hours, the FDA changed the drug classification of magnesium sulfate from category A to category D in its labeling [2]. This is unfortunate since magnesium sulfate has been used by obstetricians for many decades and certainly in many thousands of patients the exposure was prolonged. It should be realized not only were the abnormal findings self-limiting but that the investigations quoted by the FDA [25]–[28], as well as others [29],[30], had very small patient populations (usually less than 3–4 infants) and this would make the conclusions of those studies uncertain at best. There is no denominator to these studies. Is the incidence 2% or is it 0.002%?

It should also be remembered that magnesium sulfate has been used to treat millions of patients over the years who have had preeclampsia, preterm labor, and more recently for neuroprotection in fetuses destined to be delivered preterm. Demineralization and fracture has not been an issue in any of the studies in this area [3],[14],[17],[30]. Therefore it is important that physicians continue to use magnesium sulfate in these appropriate categories without fear of neonatal osteopenia or fracture. Indeed the majority of the patients in various case reports where fractures and osteopenia were reported received treatment with magnesium for 5 to 11 weeks. In addition, the majority of patients had multifetal gestations (particularly higher order multiples) and had been at extensive bed rest for much of the pregnancy. Therefore, complicating the findings of bone demineralization in the mother and/or newborn, have been other factors such as prolonged immobilization as noted above. In addition treatment in those patients with Heparin to prevent thromboembolism as well as the increased calcium demands of multifetal gestations may contribute to osteopenia and fractures. It is difficult to ascribe to magnesium alone the changes in the neonates noted in case reports and case series by the FDA [2]. More comforting is the long-term outcome of very premature infants treated or untreated with magnesium sulfate. Doyle et al [31] published results from the ACTOMgSO_4_ study which was carried out in 16 centers with over a thousand patients treated and untreated. Over 850 children in the study had a follow up of 6–11 years and there were no differences in the neurological, behavioral, growth or functional outcome between the two groups.

Equally important when we are trying, as physicians to reduce adverse effects of certain therapies; we must always remember there is the risk to the mother/fetus/newborn of not treating. The patients who received long-term therapy with magnesium sulfate included in many of the study reports [21]–[26] were women who were treated over many weeks for a good reason: principally because multifetal gestations at 22–26 weeks who were at risk for severe neonatal neurologic morbidity and death due to extreme low birth weight developed PTL. Those pregnancies that had successful long treatment episodes meant that each fetus gained 8 to 12 weeks on the average (from 22 up to 34 weeks for example). While it can be argued that patients were not in “true” preterm labor (as the obstetric data in each case was not fully expressed), certainly the treating physicians felt that the risk of delivery was high and that therapy with magnesium sulfate was necessary to prolong gestation and prevent neonatal demise or severe neurologic morbidity. While The American Congress of Obstetrics and Gynecology has noted that treatment with magnesium sulfate beyond 48–72 hours is “unindicated” [3], practitioners who are trying to prevent a neonatal death due to extreme prematurity continue to use long term magnesium sulfate in this small group of women as it is their only therapeutic option.

## 6. Overall Assessment

In the assessment of risk and benefit to the mother and fetus of magnesium sulfate tocolysis, the drug appears to be effective in appropriate dosing (6 gm bolus; 3 gm per hour or greater for maintenance) and may have a use in certain patients as long term maintenance therapy (multiple gestations, advanced cervical dilation, failure of oral agents with recurrent PTL). The risk to the mother is low (∼ 2%) for serious side effects, but careful monitoring of the patient is recommended. The benefits to the baby include neuroprotection and extending the gestational age before delivery. The potential harm to the baby is with prolonged parenteral use of IV magnesium sulfate and involves reversible bone demineralization. The mother should be apprised of the risks and benefits and allowed to make an informed decision. Certainly when it is used, the risk of neonatal osteoporotic changes needs to be disclosed and in our opinion patients should sign a disclaimer that they understand the long term use of magnesium sulfate is experimental and not supported by Level I evidence. Of course, the reason that long term use is not supported by randomized clinical trials is that this clinical circumstance is so infrequent when compared to the therapy with magnesium sulfate for neuroprotection, treatment for preeclampsia [32] and the short term treatment for acute preterm labor. It is certain that prospective studies on long term infusion of magnesium will not be carried out. Certainly it is incumbent on physicians to make certain that the patient truly needs magnesium sulfate suppression of contractions for extended periods of time and of course, use the lowest possible dose of magnesium sulfate in each case.

## 7. Conclusion

Prevention of PTD involves much more than a decision to use a particular drug to attempt to inhibit PTL. Multiple interventions are involved including: risk identification (fetal fibronectin, cervical length ultrasonography), interventions to reduce the occurrence of PTL (17 P), early detection of PTL when it occurs (patient education and awareness), effective treatment of acute PTL (tocolytic medication, pessaries [33]) , interventions that reduce or eliminate the consequences of PTD when these steps fail (corticosteroids, magnesium sulfate neuroprotective effect) and maintenance interventions to limit recurrent PTL. Each of these steps requires a clinician to weigh risks and benefits. It seems apparent that some patients will need extended use of magnesium sulfate in order to achieve a better overall outcome. Such strong pronouncements by respected groups such as ACOG and the FDA exert a powerful deterrent to the obstetrician trying to make a balanced decision about his/her patient. A more effective strategy would be to properly present the risks and benefits in a reality based assessment of the entire approach to the problem of preventing PTD. In the hypothetical patient with twins at 24 weeks, 3 cm dilated, presenting in PTL, please do more than “recommend against prolonged use of magnesium sulfate to stop preterm labor due to bone changes in exposed babies”.

## References

[1] (2012). ACOG Practice Bulletin No. 127 Management of Preterm Labor.

[2] U.S. Food and Drug Administration Drug Safety Communication (2013). FDA recommends against prolonged use of magnesium sulfate to stop pre-term labor due to bone changes in exposed babies.

[3] ACOG Committee Opinion, No. 573 (2013). Magnesium Sulfate Use in Obstetrics. Obstet Gynecol.

[4] Vintzileos AM (2009). Evidence-Based Compared With Reality-Based Medicine in Obstetrics. Obstet Gynecol.

[5] Evidence-Based Medicine Working Group (1992). Evidence-Based Medicine: A New Approach to Teaching the Practice of Medicine. JAMA.

[6] Sackett DL (1997). Evidence-Based Medicine. Seminar Perinatol.

[7] Steer CM, Petrie RH (1977). A comparison of magnesium sulfate and alcohol for the prevention of premature labor. Am J Obstet Gynecol.

[8] Elliott JP (1983). Magnesium sulfate as a tocolytic agent. Am J Obstet Gynecol.

[9] Elliott JP, Lewis DF, Morrison JC (2009). In Defense of Magnesium Sulfate. Obstet Gynecol.

[10] Ma L (1992). Magnesium sulfate in the prevention of preterm labor (translation). Chung Hua I Hsveh Chin Taipla.

[11] Fox MD, Allbert JR, McCaul JF, Martin RW, McLaughlin BN, Morrison JC (1993). Neonatal morbidity between 34 and 37 weeks gestation. J Perinatol.

[12] Cox SM, Sherman ML, Leveno KJ (1990). Randomized investigation of magnesium sulfate for prevention of preterm birth. Am J Obstet Gynecol.

[13] Elliott JP (1990). Letter to the editor- Sub therapeutic doses of magnesium sulfate do not inhibit preterm labor. Am J Obstet Gynecol.

[14] Mercer BM, Merlino AA (2009). Magnesium Sulfate Preterm Labor and Preterm Birth. Am J Obstet Gynecol.

[15] Hung J, Tsai M, Yang B (2005). Maternal Osteoporosis After Prolonged Magnesium Sulfate Tocolysis therapy: A Case Report. Arch Phys Med Rehabil.

[16] Conde-Agudelo A, Romero R (2009). Antenatal magnesium sulfate for the prevention of cerebral palsy in preterm infants less than 34 weeks gestation. Am J Obstet Gynecol.

[17] ACOG Committee Opinion No. 455 (2010). Magnesium sulfate before anticipated preterm birth for neuroprotection.

[18] Scott JR (2009). Magnesium Sulfate for neuroprotection. What do we do now?. Obstet Gynecol.

[19] Reeves SA, Gibbs RS, Clark SL (2011). Magnesium for fetal neuroprotection. Am J Obstet Gynecol.

[20] Mittendorf R, Covert R, Bowman J (1997). Is tocolytic magnesium sulfate associated with increased total pediatric mortality. Lancet.

[21] Crowther CA, Hiller JE, Doyle LW, Haslam RR (2003). Effect of magnesium sulfate given for neuroprotection before preterm birth: a randomized controlled trial. JAMA.

[22] Farkouh LJ, Thorp JA, Jones PG, Clark RH, Knox GE (2001). Antenatal magnesium exposure and neonatal demise. Am J Obstet Gynecol.

[23] Mittendorf R, Dambrosia J, Pryde PG (2002). Associationbetween the use of antenatal magnesium sulfate in preterm labor and adverse health outcomes in infants. Am J Obstet Gynecol.

[24] Elliott JP, Morrison JC The Evidence Regarding Maintenance Tocolysis. Obstetrics and Gynecology International.

[25] Yokoyama K, Takahashi N, Yada Y (2010). Prolonged maternal magnesium administration and bone metabolism in neonates. Early Hum Dev.

[26] Wedig KE, Kogan J, Schorry EK (2006). Skeletal demineralization and fractures caused by fetal magnesium toxicity. J Perinatol.

[27] Malaeb SN, Rassi A, Haddad MC (2001). Bone mineralization in newborns whose mothers received magnesium sulfate for tocolysis of premature labor. Pediatr Radiol.

[28] Kaplan W, Haymond MW, Mckay S, Karaviti LP (2006). Osteopenic effects of magnesium sulfate in multiple pregnancies. J Pediatric Endocrinology and Metabolism.

[29] Nassar AH, Sakhel K, Maarouf H (2006). Adverse maternal and neonatal outcome of prolonged course of magnesium sulfate tocolysis. Acta Obstet Gynecol Scan.

[30] Schanier RJ, Smith LG, Burns PA (1997). Effects of long-term maternal intravenous magnesium sulfate therapy on neonatal calcium metabolism and bone mineral content. Gynecol Obstet Invest.

[31] Doyle LE, Anderson PJ, Haslam R (2014). School-age outcomes of very preterm infants after antenatal treatment with magnesium sulfate vs. placebo. JAMA.

[32] ACOG Practice Bulletin No. 33 (2002). Diagnosis and management of preeclampsia and eclampsia. American College of Obstetricians and Gynecologists. Obstet Gynecol.

[33] Keys S, Elliott JP (1997). The therapeutic use of vaginal pessary as adjunctive treatment in patients with multiple gestations presenting with preterm labor and low fetal station. J Reprent Med.

